# An immunological approach of sperm sexing and different methods for identification of X- and Y-chromosome bearing sperm

**DOI:** 10.14202/vetworld.2017.498-504

**Published:** 2017-05-09

**Authors:** Shiv Kumar Yadav, Dharmendra Kumar Gangwar, Jarnail Singh, Chiranjeev Kumar Tikadar, V. Vinoth Khanna, Sudha Saini, Sunny Dholpuria, Prabhat Palta, Radhey Shyam Manik, Manoj Kumar Singh, Suresh Kumar Singla

**Affiliations:** Embryo Biotechnology Laboratory, Animal Biotechnology Centre, ICAR-National Dairy Research Institute, Karnal - 132 001, Haryana, India

**Keywords:** differentially expressed proteins, H-Y antigen, sex specific proteins, sperm identification, sperm sexing

## Abstract

Separation of X- and Y-chromosome bearing sperm has been practiced for selection of desired sex of offspring to increase the profit in livestock industries. At present, fluorescence-activated cell sorter is the only successful method for separation of X- and Y-chromosome bearing sperm. This technology is based on the differences in DNA content between these two types of sperm and has been commercialized for bovine sperm. However, this technology still has problems in terms of high economic cost, sperm damage, and lower pregnancy rates compared to unsorted semen. Therefore, an inexpensive, convenient, and non-invasive approach for sperm sexing would be of benefit to agricultural sector. Within this perspective, immunological sperm sexing method is one of the attractive choices to separate X- and Y-chromosome bearing sperm. This article reviews the current knowledge about immunological approaches, viz., H-Y antigen, sex-specific antigens, and differentially expressed proteins for sperm sexing. Moreover, this review also highlighted the different methods for identification of X- and Y-sperm.

## Introduction

The possibility to control the sex of offspring in farm animals is a topic of great interest for researchers of agriculture sector. Controlling the sex ratio entails direct returns in the livestock sector, allowing improved management of food production, animal welfare improvement, faster genetic selection, and a decrease of environmental impact [1]. Separation of X- and Y-sperm for pre-selection of the desired sex is economically important in livestock production, which allows the livestock sector to produce the optimal proportion of males and females [2]. Moreover, predetermination of sex can reduce the management cost thorough selective management of superior bulls or cows [3]. In mammals, sex determination is strictly chromosomal, and the sex of an offspring is entirely decided by the sperm. Male produces two types of sperm, half bearing the X-chromosome (X-sperm) and remaining half the Y-chromosome (Y-sperm) whereas the oocyte (ovum) produced by the females always carry an X-chromosome. Therefore, fertilization of an ovum by a Y-sperm produces a male (XY) and fertilization by an X-sperm produces a female (XX). In mammals, the X-sperm contain more DNA than the Y-sperm. The degree of differences varies from species to species and amounts to approximately 2.9% in human sperm [4,5], 3.8% in cattle [6,7], and as much as about 7.5% in chinchilla [8]. In addition to DNA content, other differences include the size (X-sperm Y-sperm) [9,10], surface charges on sperm (Y-sperm has a positive charge and X-sperm has a negative charge) [11] and cell surface antigens [12]. Furthermore, in a study with bull sperm, Penfold *et al*. [13] reported that Y-sperm does not swim faster than X-sperm. However, it may be distinguished from X-sperm on the basis of linearity and straightness of path.

Based on the theoretical differences, numerous methods have been reported for sorting of X- and Y-sperm. These methods include flow cytometry [14], percoll and albumin gradient centrifugation [15], swim up [16], sephadex columns [17], and H-Y antigen [18]. At present, only flow cytometry, pioneered by Johnson *et al*. [19], has been proved to effectively sort X- and Y-sperm [20]. Sperm sorting based on DNA differences by using flow cytometry has been largely accepted as a major breakthrough in the reproduction technology [21]. This technology has progressed sufficiently to allow commercial use only in the bovine species [22,23]. However, several publications on semen sexing using flow cytometry are being reported on other species to allow commercial use [24-29]. However, sex-sorted sperm using flow cytometric technique still has difficulties in terms of sperm damage, high economic cost, complexity of operation, and lower pregnancy rates than the traditional semen [30-33]. These problems prompted to establish efficient, inexpensive, convenient, and non-invasive approaches for sperm sorting. In this regard, immunological method for sperm sexing would be of benefit to agricultural sector.

## Basis of Immunological Sperm Sexing

The observed genomic DNA differences among X- and Y-sperm across different species led to the possibility that these DNA differences might result in the protein differences as well. In recent past, Chen *et al*. [34] reported 31 differentially expressed genes. Among these, 27 were up-regulated in X-sperm and 4 in Y-sperm. Differential expression of genes between X- and Y-sperm may lead to phenotypic variations in X- and Y-sperm. The basic concept of immunological methods for sperm sexing is based on the different proteins present on the surface of X- and Y-sperm [35]. The theory behind this concept is that if one can isolate/identify such a marker(s), then antibodies could be developed against X- and/or Y-specific surface protein(s). Subsequently, the use of magnetic bead, affinity chromatography, and sperm identification (fluorescence-activated cell sorting [FACS]) technique would provide a batch separation process for the same. However, the possibility of detecting and possibly separating a recognized cell by using specific antibodies is linked to the accessibility of antibodies to the selected protein targets [1].

## Different Approaches of Immunological Sperm Sexing

### Cell surface antigens

Numerous immunological approaches for sperm sexing have been tested without repeatable success [14,36]. Cell surface antigens specific to either X- or Y-sperm offer a potential means of separating two sperm populations [37]. H-Y antigen, a male-specific protein has attracted attention of different researchers as a possible means of discriminating Y-sperm from X-sperm [37]. H-Y antigen is found in male tissues of many mammalian species with the exception of erythrocytes and premeiotic germ cells. If the expression of H-Y antigen on the surface of these haploid cells is due to expression of the Y-chromosome, then this could be used to separate Y-sperm from X-sperm [38]. There are several conflicting reports about a possible difference between X- and Y-sperm in expressing H-Y antigen. Several researchers reported the preferential binding of anti-H-Y antibody to Y-sperm [39,40]. Ali *et al*. [40] identified and separated X-sperm from Y-sperm using monoclonal H-Y antibodies. They observed that specific H-Y antibody could bind to around half the sperm population. However, sex ratio of the offspring was not altered after treatment of sperm with the same antibody. Moreover, others did not find evidence for the H-Y antigen to be preferentially present on Y-sperm [41]. Furthermore, Prasad *et al*. [38] concluded that H-Y antigen was present on the cell membranes of both X- and Y-sperm under normal circumstances. Therefore, the probability of sexing mammalian semen using H-Y antibody is not appropriate.

### Sex-specific antigens

The identification of sex-specific proteins (SSP) in X- or Y-sperm would be helpful to develop an immunological method for separation of sperm. Numerous attempts to search for differences between X- and Y-sperm have been reported using two-dimensional gel electrophoresis (2-DE). However, this technique was ineffective in providing any evidence of the SSP [14,36]. However, some indirect evidence implied that differences might exist between X- and Y-sperm [42,43]. Due to lack of success of this approach, it is speculated that variations in protein composition between X- and Y-sperm might occur for a minor component of the sperm membrane with a low antigenicity or carbohydrate epitope, and membrane proteins might be below the present detection level by 2-DE [36,44]. Therefore, it prompted to search for an attractive alternative that can be used to detect the low level of membrane protein of sperm. In this regard, an immunological approach may offer an alternative and efficient technique for searching SSPs [45].

This approach is based on the hypothesis that SSPs are evolutionarily more highly conserved than non-SSPs [46]. Blecher *et al*. [46] raised antibodies to non-SSPs in rabbit and used an affinity procedure to remove non-SSPs and enrich for SSPs. Afterward, they obtained purified SSPs using column chromatography. Development of antibodies against these SSPs appears to bind to sex-chromosome-specific proteins on the sperm membrane. On the basis of this experiment, they opined that X- and Y-sperm do carry different proteins on their cell surfaces and it may be possible to develop an immunological-based approach for sperm separation. In another study, Sang *et al*. [47] attempted to identify sex-specific antibodies in rabbit antisera against bovine sex-sorted sperm, and further purified through immuno-neutralization with excess of sex-sorted Y- or X-sperm, respectively, to remove non-sex specific antibodies and enrich sex-specific antibodies. Subsequently, the purified rabbit sera with enriched sex-specific antibodies were screened for sex-specific antibodies by immunofluorescence staining and flow cytometry. In this study, they confirmed sex-specific antibodies in the putative XSSAb, and a SSP (about 30 kDa) also was captured by the antisera. Finally, they advocated that SSPs might be present on the X-sperm surfaces, and this may offer some assistance to exploit an immunological procedure for sexing sperm. Sex-specific gene, sex-determining region Y (SRY) is specifically localized in Y-chromosome and it encodes SRY protein. In Y-sperm, both transcript and protein have been reported [48]. However, there is no report available whether this protein is associated with sex selection [49].

### Some Recent Proteomics Approaches Based on Differentially Expressed Proteins

The use of proteomics in sperm biology plays an important role invisible in any other tissue/cell type beside blood. Sperm can be obtained in high purity and concentrations. Therefore, it is arguably the best cell type for proteomic analysis [50]. The advantage of proteomics approach is that the method is capable of generating large amounts of significant data in a relatively short time frame and gives us a snapshot of proteins present within the sperm. In the recent past, the differences in numerous proteins between X- and Y-sperm have been investigated by different research groups to find the differential proteins [1,51,52]. These proteins may provide a new way to separate X- and Y-sperm by using immunological methods. The application of proteomic approach in this field leads to unparalleled progress in the detection of sperm protein constituents, such as kinases, transmembrane proteins and chaperones never previously recognized, thus provides promising means to answer biological questions related to sperm [50].

De Canio *et al*. [1] performed a comparative proteomic study by nano ultra-performance liquid chromatography-tandem mass spectrometry to characterize pooled samples of sexed bovine semen. The protein profiles of X- and Y-sperm have shown differential expression of proteins. In this study, they found 17 differentially expressed proteins (Table-1). Among these proteins, 15 were up-regulated in X-sperm and 2 in Y-sperm. These proteins were found to be directly associated with the cytoskeletal structures of flagellum, glycolytic enzymes and calmodulin. In another study, 42 significant protein spots were differentially expressed in X- and Y-sperm of bull. 16 of them were relevant to energy metabolism, cytoskeletal structure, stress resistance, and the activity of serine proteases [52]. These differentially expressed proteins may affect the sperm functions, phenotype, interaction between sperm and oocyte, and development of the zygotic embryo [49,52]. These differentially expressed proteins can be used as tentative molecular markers to differentiate X- and Y-sperm and sorting as well. Sperm acrosome membrane-associated protein 1 (SACA1) is the major component of bovine seminal plasma and involved in sperm capacitation [53]. This protein has been detected in acrosome of human sperm with a higher concentration in the equatorial segment [54]. Furthermore, SACA1 was clearly shown to be a differentiation antigen in humans and expressed exclusively in germ cells during acrosomal biogenesis [55]. Based on acrosomal localization and antigenic properties of SACA1, this protein may be a promising candidate for an antibody-based separation of sperm [1]. In future, differentially expressed proteins localized in sperm membrane may be promising for the development of new technology for sperm sexing and identification of X- and Y-sperm.

**Table-1:** Differentially expressed proteins in sexed sperm (adapted and modified from De Canio *et al*. [1]).

Differentially expressed proteins	Up-regulated		Biological role

	X-sperm	Y-sperm	
Outer dense fiber protein 1	Yes	……	Structural function
Outer dense fiber protein 2	Yes	……
Outer dense fiber protein 3	Yes	……
A kinase anchor protein 3	Yes	……
Tubulin alpha 3	Yes	……
Tubulin alpha 8	……	Yes
Tubulin beta 4A	Yes	……
Tubulin beta 2B	……	Yes
Tubulin beta 4B	Yes	……

Glyceraldehyde 3 phosphate dehydrogenase	Yes	……	Glycolytic enzyme
L-lactate dehydrogenase A	Yes	……
Triosephosphate isomerase	Yes	……

Seminal plasma protein PDC 109	Yes	……	Sperm capacitation and sperm tail beating
Calmodulin	Yes	……
Sperm acrosome membrane associated protein 1	Yes	……

L-asparaginase	Yes	……	Amino acid catabolism

Glyceraldehyde 3 phosphate dehydrogenase testis specific	Yes	……	Sperm motility regulation

### Confirmation of X- and Y-sperm enrichment

Following sperm sorting, it is desirable to obtain information about the purity of the sperm. Several studies have been hampered by the lack of a reliable and reproducible method for estimating the percentage of X- and Y-sperm in different fractions. Different methods for identification of X- and Y-sperm are as follows:

#### Quinacrine mustard staining

Quinacrine mustard staining produces very intense fluorescence to certain regions of chromosome [56]. Previously, quinacrine staining was used to verify X- or Y-sperm enrichment, in which the putative Y-chromosome bearing sperm exhibit a fluorescent spot or F body, and the putative X-chromosome bearing sperm remain unstained [57]. However, quinacrine produces false positive and false negative results in interphase cells and several studies have shown that this technique can produce misleading and imprecise results with human sperm [58,59]. In another study, Pearson *et al*. [60] performed quinacrine staining in several mammals including humans. They reported that quinacrine fluorescence is not universal property of all mammalian Y-chromosome and fluorescence intensity found on the human Y-chromosome is only present in the African great apes and man. Therefore, it is considered an inappropriate approach for selection of sperm for most mammalian species.

#### Flow cytometry

At present, separation of X- and Y-sperm by flow cytometry is the only proven effective method for sexing viable mammalian sperm. Following sperm sorting, the putative X- and Y-sperm populations are re-stained and passed back through the cell sorter so that the purity of the populations can be readily determined [7,61-63]. However, the validation by flow cytometric re-measurement of the sexed sperm relies on the same instrument which produced the original sperm separation [64]. Therefore, a convenient validation method is required for sorting sperm with either X- or Y-chromosomes in each species.

### Polymerase chain reaction (PCR) and fluorescence in situ hybridization (FISH)

With the introduction of PCR and FISH, it has now become possible to accurately identify the X- and Y-sperm and this has opened the way for assessment of sorting purity of different sperm sexing methods [59]. Specific DNA sequences on X- and Y-sperm have been reported which can be used to identify the sex of individual sperm and sex ratios of sperm in semen sample [65,66]. Accurate determination of the sex ratio using single sperm PCR necessitates analysis of a large number of individual sperm. However, sex ratio of semen can be determined more simply and accurately by quantitative real-time PCR (qPCR) [67]. Khamlor *et al*. [68] determined the sperm sex ratio in bovine semen by using multiplex real-time PCR. In this experiment, they have used specific sequences of bovine proteolipid protein gene located on the X-chromosome and another for SRY located on the Y-chromosome. They found accurate assessment of the sperm sex ratio in both sorted and unsorted semen. In another study, Tan *et al*. [69] determined the sex of bovine sperm using SYBR^®^ Green qPCR. In this study, two sets of primers zinc finger protein X (ZFX), specific sequences for X-chromosome and another Y-chromosome-specific sequence SRY was used. In bovine genome, both ZFX and SRY genes are exist as single copy number and commonly used as a DNA marker in gender determination [69]. PCR is very specific and highly sensitive technique to identify the X- and Y-sperm. However, its application to populations of cells is of limited use in the assessment of sex selection methods. Moreover, it is labor intensive to be used for screening large number of individual sperm [59].

Single and double label FISH can be used for the direct visualization of sex chromosomes in individual sperm. FISH precisely identifies the sex chromosome of individual sperm using specific probes conjugated with fluorescence molecule for the X- and Y-sperm [59]. The application of double FISH using X- and Y-chromosome-specific probes has allowed a more accurate assessment than single label FISH [70]. The main advantage of FISH compared to flow cytometry reanalysis and single cell PCR evaluation is that it is highly qualitative and quantitative [71]. However, the major problem with FISH is the degree of condensation of the DNA in sperm which creates difficulties in accessing specific hybridization sites. During FISH, the nuclei of the sperm must be decondensed to allow the access of DNA probes to the sperm chromatin [72].

### Raman microspectroscopy

Recently, De Luca *et al*. [73] reported discrimination of X- and Y-bovine sperm based on Raman spectroscopy. They compared the Raman spectra of X- and Y-sperm from three different bulls and demonstrated that a spectroscopic signature, combination of the spectral component in the sperm (DNA, protein, lipids, etc.), can be used to identify the differences between X- and Y-sperm. The nucleus reveals the main biochemical differences between X- and Y-sperm. The most evident differences between X- and Y-sperm can be found in the peak at 726, 785 and 1581/cm (assigned to nucleic acids and DNA backbone) and a sharp peak at 1095/cm is indicative of the PO_2_^−^ backbone. An increased intensity of the peaks was observed in X-sperm compared to Y-sperm indicating an overall DNA content [73,74]. Moreover, spectrum of the acrosomal vesicle shows higher intensity of the peaks corresponding to proteins, Amide I (1600-1680/cm), Amide III (around 1200-1300/cm), and lipids (C-H vibration at 1480/cm) [73]. The tail spectrum is characterized by a peak around 751/cm previously assigned to mitochondria [75]. Raman peak positions and relative intensities are consistent in the three nuclear regions, viz., acrosomal, middle, and neck regions. The main variations of Raman peaks were observed due to DNA content together with the sex membrane proteins [73]. De Luca *et al*. [73] advocated that Raman spectroscopy is a promising candidate for the development of a highly efficient and non-invasive technique for sperm sexing.

## Conclusion and Future Perspective

Numerous methods have been reported to separate X- and Y-chromosome bearing sperm. However, the common underlying problem from these methods has been the lack of reproducibility. At present, FACS is the only proven effective method for sexing viable mammalian sperm. However, sexing of sperm by FACS technique still has problems in terms of high economic cost and sperm damage. Therefore, an economical, convenient, and non-invasive approach such as immunological methods for sperm sexing would be of benefit to agricultural sectors. In this regard, differentially expressed proteins present on the membrane of X- or Y-sperm may be promising for the development of new technology for semen sexing and identification of X- and Y-sperm.

## Authors’ Contributions

SKY conceptualized, designed and wrote the manuscript. DKG and JS contributed in manuscript writing and design. CKT and VVK contributed in literature collection and reviewed the manuscript. SS and SD made critical comments and helped in revising the manuscript. PP edited and RSM and MKS made critical comments on manuscript. SKS conceptualized, designed, made critical comments on revised manuscript and edited the manuscript for final version.
